# Systematic review and meta-analysis: the relationship between the *Helicobacter pylori dupA *gene and clinical outcomes

**DOI:** 10.1186/1757-4749-2-13

**Published:** 2010-10-31

**Authors:** Seiji Shiota, Osamu Matsunari, Masahide Watada, Katsuhiro Hanada, Yoshio Yamaoka

**Affiliations:** 1 Department of Environmental and Preventive Medicine, Oita University Faculty of Medicine, 1-1 Idaigaoka, Hasama-machi, Yufu-City, Oita 879-5593, Japan; 2 Department of General Medicine, Oita University Faculty of Medicine, 1-1 Idaigaoka, Hasama-machi, Yufu-City, Oita 879-5593, Japan; 3 Department of Medicine-Gastroenterology, Baylor College of Medicine and Michael E. DeBakey Veterans Affairs Medical Center, 2002 Holcombe Blvd. Houston, Texas 77030 USA

## Abstract

**Background:**

In 2005, the first disease-specific *Helicobacter pylori *virulence factor that induced duodenal ulcer and had a suppressive action on gastric cancer has been identified, and was named duodenal ulcer promoting gene (*dupA*). However, the importance of the *dupA *gene on clinical outcomes is conflicting in subsequent studies. The aim of this study was to estimate the magnitude of the risk for clinical outcomes associated with *dupA *gene.

**Methods:**

A meta-analysis of case-control studies which provided raw data on the infection rates with the *dupA*-positive *H. pylori *detected by polymerase chain reaction was performed.

**Results:**

Seventeen studies with a total of 2,466 patients were identified in the search. Infection with the *dupA*-positive *H. pylori *increased the risk for duodenal ulcer by 1.41-fold (95% confidence interval [CI], 1.12-1.76) overall. Subgroup analysis showed that the summary odds ratio (OR) was 1.57 (95% CI, 1.19-2.06) in Asian countries and 1.09 (95% CI, 0.73-1.62) in Western countries. There was no association between the presence of the *dupA *gene and gastric cancer and gastric ulcer. Publication bias did not exist.

**Conclusion:**

Our meta-analysis confirmed the importance of the presence of the *dupA *gene for duodenal ulcer, especially in Asian countries.

## Background

*Helicobacter pylori *(*H. pylori*) infection is now accepted as the major cause of chronic gastritis. Several epidemiological studies have shown that *H. pylori *infection is also linked to the severe gastritis-associated diseases, including peptic ulcer and gastric cancer (GC) [1]. In 1994, the International Agency for Research on Cancer categorized *H. pylori *infection as a definite group I carcinogen [2]. The infection remains latent in the majority of infected patients, with only approximately 20% of infected individuals developing severe diseases. One possible reason for the varying outcomes of *H. pylori *infection relates to differences in the virulence of *H. pylori *strains in addition to host, environmental, and dietary factors.

Several *H. pylori *virulence factors associated with peptic ulcer and GC have been reported, including *cagA*, *vacA*, *babA *and *oipA *[1,3-6]. Lu *et al*. [7] described a novel virulence factor, duodenal ulcer promoting gene (*dupA*), which encompassed both *jhp0917 *and *jhp0918 *located in the plasticity region of the *H. pylori *genome. Interestingly, the *dupA *gene is homologous to *virB4*, a gene encoding a component protein of the type IV secretion system (TFSS) in *Agrobacterium tumefaciens*. They reported that infections with *dupA*-positive strains increased the risk for duodenal ulcer (DU) but were protective against gastric atrophy, intestinal metaplasia and GC in the Japanese, Korean and Columbian subjects. Intriguingly, *dupA *is the first genetic factor of *H. pylori *to be associated with differential susceptibility to DU and GC, and thus it could be considered as a disease specific virulence marker. The pathogenic mechanism of *dupA *appears to involve the induction of interleukin (IL)-8 production in the antrum, leading to antrum-predominant gastritis, a well-recognized characteristic of DU [7].

However, the role of *dupA *was controversial subsequently since several studies were unable to reproduce the observation in other population including Japan [8-12]. Although Hussein recently reported the systematic review which proved the high prevalence of *dupA*-positive *H. pylor*i in patients with DU [13], he determined the conclusion based on simple combined data but not meta-analysis (e.g., he did not show the meta-analysis model, heterogeneity, publication bias). In this study, we aimed to examine the relationship between the *dupA *gene and clinical outcomes based on the method of meta-analysis.

## Methods

A literature search was performed using the PubMed databases for articles published through August 2010, using the following text words: 1) *dupA*, and 2) *pylori *or *Helicobacter*. We did not include abstract alone or unpublished articles.

### Inclusion Criteria

The following criteria were applied to select fully published case-control studies examining the relationship between the *dupA *gene and clinical outcomes (gastritis, gastric ulcer [GU], DU, and GC) in adult population; the presence of *dupA *gene was examined by polymerase chain reaction (PCR), or PCR plus dot blot; original articles published in English. Studies were excluded if raw data were not presented. When it appeared that the same subjects were presented in multiple reports, the earliest paper was selected. All potentially relevant articles were reviewed by two investigators (S.S and Y.Y) independently and disagreement was resolved by discussion.

### Data Extraction

Data were extracted from each study by investigators independently and entered into a computerized database. The information retrieved covered countries where the study was performed, characteristics of cases and controls, method for detection of the *dupA *gene, number of subjects, the *dupA *status according to clinical outcomes. Three studies examined the prevalence of the *dupA *gene in several countries [7,8,11], thus these data in each country was entered in separate sheet as an independent study.

### Statistical analysis

Summary odds ratios (ORs) and 95% confidence intervals (CIs) were calculated from the raw data. The Mantel-Haenszel method was used to test for statistical heterogeneity. When statistical heterogeneity was noted, the proportion of the total heterogeneity variance was calculated using a fixed-effects model from each study to guide the search for sources of methodologic and clinically important variables. To exclude any possible influence of a single study, we performed a sensitivity analysis to evaluate whether the exclusion of any single study substantial altered the magnitude or statistical result of the summary estimate. Publication bias was assessed by funnel plots and regression test by Egger *et al *[14]. P value of < 0.05 was considered as statistically significant in all meta-analyses. All analyses were performed using Comprehensive Meta-analysis software (version 2, Biostat, Englewood, NJ).

## Results

The literature searches generated 17 potentially relevant citations. Of these, six articles were excluded (3 were review articles [13,15,16], one was not case-control study [17], one was in vitro study [18], and one was following study [19]), therefore 11 articles met the inclusion criteria. Three articles included several countries (three in Lu *et al*, four in Argent *et al*, and two in Schmidt *et al*). Although Schmidt *et al*. examined the *dupA *status in five countries [11], data from only two countries was case-control. In this study, data from different countries in one article was considered as separate studies (data); therefore, seventeen studies with a total of 2,466 patients met the inclusion criteria (Table 1). Among these studies, there was no age- and sex-matched case-control study. The *dupA *status was evaluated by PCR technique except two studies which performed PCR and dot blot technique [12,20]. Multiple primer pairs were used in 11 studies [8,10-12,20-22]. The *dupA *status in seven studies was determined based on the presence both of *jhp0917 *and *jhp0918 *(*dupA*-positive only if both *jhp0917 *and *jhp0918 *were positive) [7,9,22-24].

**Table 1:** List of studies included in the meta-analysis

			Gastritis			Duodenal ulcer			Gastric ulcer			Gastric cancer		
Authors	Country	Reference	Positive	all		positive	all		positive	all		positive	all	
Lu	Japan	7	7	50	14.0%	11	30	36.7%	13	50	26.0%	3	30	10.0%
Lu	Korea	7	2	30	6.7%	24	65	36.9%	5	30	16.7%	3	50	6.0%
Lu	Colombia	7	15	40	37.5%	22	45	48.9%	12	30	40.0%	6	50	12.0%
Arachchi	India	20	16	70	22.9%	36	96	37.5%						
Argent	South Africa	8	11	15	73.3%	12	13	92.3%				16	18	88.9%
Argent	Belgium	8	29	76	38.2%	20	40	50.0%				10	19	52.6%
Argent	USA	8	9	20	45.0%	9	21	42.9%	2	3	66.7%			
Argent	China	8	3	12	25.0%	2	11	18.2%	2	5	40.0%	1	1	100.0%
Douraghi	Iran	9	34	68	50.0%	15	30	50.0%	9	23	39.1%	20	36	55.6%
Gomes	Brazil	10	133	144	92.4%	110	126	87.3%				71	81	87.7%
Hussein	Iraq	21	5	29	17.2%	9	15	60.0%	2	5	40.0%			
Pacheco	Brazil	23	18	29	62.1%	13	20	65.0%	14	24	58.3%			
Zhang	China	24	51	133	38.3%	46	101	45.5%	11	47	23.4%	19	79	24.1%
Schmidt	Sweden	11	13	20	65.0%	7	11	63.6%				13	21	61.9%
Schmidt	China	11	15	52	28.8%	10	16	62.5%				12	22	54.5%
Nguyen	Japan	12	23	78	29.5%	17	62	27.4%	17	59	28.8%	10	34	29.4%
Yeh	Taiwan	22	24	94	25.5%	13	57	22.8%	6	30	20.0%			

### The association between the *dupA *status and duodenal ulcer

**Figure 1:**
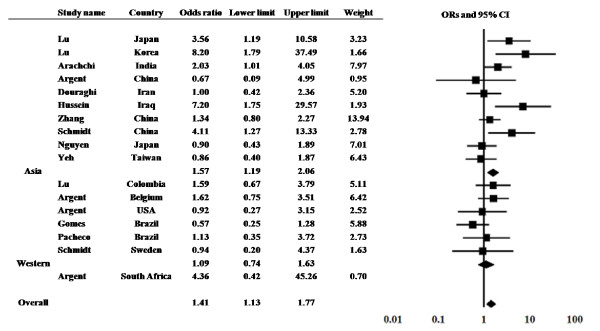
**The results of meta-analysis for the risk of duodenal ulcer in *dupA*-positive *H. pylori *infection**. Odds ratios (ORs) and their 95% confidence intervals (CI) in summary and for each study are presented with weighting in a fixed-effect model.

The prevalence of the *dupA *gene in DU patients was examined in 17 studies from 13 countries (Figure 1). The prevalence of the *dupA *gene ranged from 18.2% to 92.3% in DU patients and 6.7% to 92.4% in controls. Among 17 studies, significantly higher prevalence of the *dupA *gene in DU compared with control was found in five studies [7,11,20,21].

The overall prevalence of the *dupA *gene was 49.5% (376 of 759) in DU and 42.5% (408 of 960) in controls, yielding an estimate OR of 1.33 (95% CI, 1.09-1.60). Summary OR in fixed-effect model was 1.41 (95% CI, 1.12-1.76). However, the test of heterogeneity was significant among these studies (Q = 28.15 with d.f. = 16, p = 0.03), suggesting the existence of either methodologic or clinical heterogeneity. By exploring the sources of heterogeneity, we found that the study by Gomes *et al*. [10] showed the larger differential in the prevalence of the *dupA *gene compared with other studies. They reported the prevalence of the *dupA *gene was 87.3% in DU and 92.4% in control. In addition, the study by Lu *et al*. in Korea showed the lower prevalence of the *dupA *gene especially in control (6.7%) [7]. Sensitivity analysis excluding these 2 studies showed a similar OR of 1.46 with a 95% CI of 1.15-1.85 and the test of heterogeneity was no longer statistically significant (Q = 18.05 with d.f. = 14, p = 0.20). Publication bias did not exist (intercept, 1.28; p = 0.17).

Subgroup analysis was also performed by two areas (Asian or Western countries). The study in South Africa [8] was not included in both. The prevalence of the *dupA *gene was 37.9% (183 of 483) in DU and 29.2% (180 of 616) in controls in Asian countries. It was 68.8% (181 of 263) in DU and 66.0% (217 of 329) in controls in Western countries. Summary OR was 1.57 (95% CI, 1.19-2.06) in Asian countries and 1.09 (95% CI, 0.73-1.62) in Western countries. When the study by Gomes *et al*. was excluded, it was 51.8% (71 of 137) in DU and 45.4% (84 of 185) in controls in Western countries (OR = 1.34 with a 95% CI, 0.85-2.13).

### The association between the *dupA *status and gastric cancer

**Figure 2:**
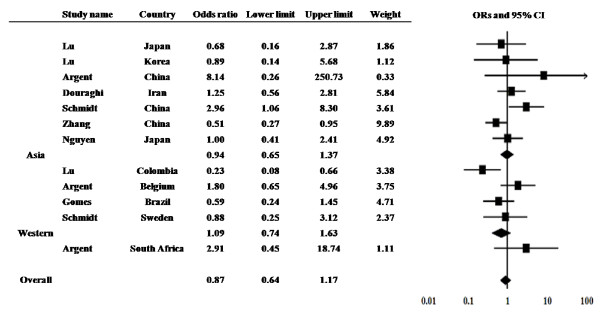
**The results of meta-analysis for the risk of gastric cancer in *dupA*-positive *H. pylori *infection**.

The prevalence of the *dupA *gene in GC patients was examined from 12 studies from 9 countries (Figure 2). The prevalence ranged from 6.0% to 100% in GC patients and 6.7% to 92.4% in controls. Among 12 studies, significantly higher prevalence in GC compared with control was found in two studies [7,11].

The overall prevalence of the *dupA *gene was 41.7% (184 of 441) in GC and 46.8% (336 of 718) in controls, yielding an estimate OR of 0.80 (95% CI, 0.63-1.02). Summary OR in fixed-effect model was 0.88 (95% CI, 0.66-1.17). However, significant heterogeneity existed among these studies (Q = 21.44 with d.f. = 12, p = 0.04). The study in Colombia by Lu *et al*. [7] showed the larger differential in the prevalence of the *dupA *gene when compared with other studies. They reported the prevalence of the *dupA *gene was 6.0% in GC and 6.7% in control. Sensitivity analysis excluding this study showed a similar OR of 0.98 with a 95% CI of 0.72-1.32 with no heterogeneity (Q = 14.73 with d.f. = 11, p = 0.19).

Subgroup analysis also did not show the significant association between GC and *dupA *both in Asian and Western countries. The prevalence of the *dupA *gene was 27.0% (68 of 252) in GC and 31.9% (135 of 423) in controls in Asian countries. The prevalence was 58.5% (100 of 171) in GC and 67.9% (190 of 380) in controls in Western countries.

### The association between the *dupA *status and gastric ulcer

**Figure 3:**
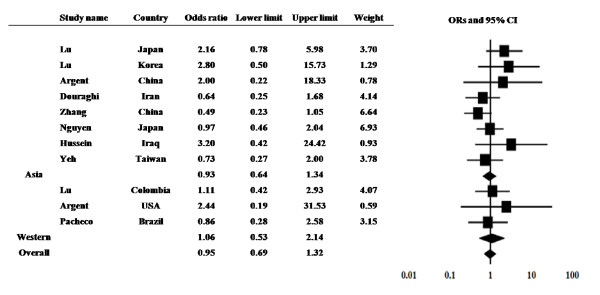
**The results of meta-analysis for the risk of gastric ulcer in *dupA*-positive *H. pylori *infection**.

The prevalence of the *dupA *gene in gastric ulcer (GU) patients was examined from 11 studies from 9 countries (Figure 3). The prevalence ranged from 16.7 to 66.7% in GU patients and 6.7 to 62.1% in controls. Among 11 studies, no study showed the significantly higher prevalence in GU compared with control.

The overall prevalence of the *dupA *gene was 30.3% (93 of 306) in GU and 32.7% (191 of 583) in controls, yielding an estimate OR of 0.90 (95% CI, 0.66-1.20). Summary OR in fixed-effect model was 0.95 (95% CI, 0.68-1.32) with no heterogeneity (Q = 10.25 with d.f. = 10, p = 0.41). Subgroup analysis also did not show the significant association between GC and the *dupA *status both in Asian and Western countries. The prevalence of the *dupA *gene was 26.1% (65 of 249) in GU and 30.2% (149 of 494) in controls in Asian countries. The prevalence was 49.1% (28 of 57) in GC and 47.2% (42 of 89) in controls in Western countries.

### The difference of prevalence in Asian and Western countries

The overall prevalence of the *dupA *gene was 31.0% (496 of 1,600) in Asian countries and 64.1% (526 of 820) in Western countries. It was significantly higher in Western countries than Asian countries (p < 0.0001). When the study by Gomes *et al*. was excluded, this trend did not change (p < 0.0001).

## Discussion

Our present meta-analysis shows that the presence of the *dupA *gene was significantly associated with DU. Although several studies failed to show the positive association between the *dupA *status and clinical outcomes, the meta-analysis confirmed the original report in 2005 [7]. Especially, the presence of the *dupA *gene was associated with DU in Asian countries; however it was not in Western countries. This difference may be due to the different prevalence of the *dupA *gene between Asian and Western countries. Furthermore, the study by Gomes *et al*. was the resource of heterogeneity due to larger difference compared with other studies. This mean the simple combined calculation such as the report by Hussein [13] is not strictly accurate to concluded although the trend was not changed. In addition, we found that the several miscount exist in the report by Hussein (e.g., he counted the mean age but not the number of subjects from the study Zhang *et al*. [24] and mistook the calculation from the study by Pacheco *et al*. [23]).

When Argent *et al*. combined Belgian and South African populations, the presence of the *dupA *gene was significantly associated with the presence of GC. There was also a non-significant trend towards an association between the *dupA *gene and DU in the combined Belgian and South African population. However, we did not include the South Africa in Western countries. It is not relevant to combine random populations since it is unclear whether the South African strains were taken from patients of European descent and recent studies confirmed that the genomic structures of some South African strains (i.e., HpAfrica2 type) were relatively different from those from the European population (i.e., HpEurope type) [25,26].

In a study examining strains from Brazilian children and adults, the prevalence of the *dupA *gene was extremely high (92%; 445/482) irrespective of clinical outcomes [10]. Interestingly, the frequency of the *dupA *gene was significantly higher in strains from children than in those from adults. *H. pylori *infection is typically acquired in childhood and persists throughout life unless treated with a combination of anti-acid and antimicrobial therapy, so it is speculated that the *dupA *gene might be lost during long-term infections in which the gastric mucosa gradually develop chronic atrophic gastritis and GC. Therefore, their results might partially support the original hypothesis that the *dupA *gene is a marker associated with gastric damage that leads to the development of gastric cancer. Therefore, we need to pay attention to the age of each disease in case-control study. For example, mean age of DU was younger than that of gastritis in the study by Zhang *et al*. (41 years old in DU vs 59 years old in gastritis) [24]. Case-control study matched age- and sex- should be performed in the future.

Overall, there are distinct geographical variations in the prevalence of the *dupA *gene, and there appears to be an association between *dupA *and DU in some populations but not in others. As Argent *et al*. [8] reported, the association of *dupA *with DU in only some populations could reflect differences in the definition or diagnosis of ulcers or in the use of drugs that either cause or heal ulcers in these populations. In addition, the discrepancy could be related to the limitation of PCR techniques for detecting the intact *dupA *gene. In some studies, only one set of primer pairs for *jhp0917 *and *jhp0918 *was used [7,9,23,24]: use of multiple primer pairs is recommended for detection of the *dupA *gene in future studies. None of the previous reports considered the frameshift mutation after position 1385 as a criterion for the presence of the *dupA *gene [7,10]. More importantly, Gomes *et al*. reported frameshift mutations in 14/86 (16%) *dupA*-positive sequenced samples [10]; a single adenine insertion after position 1426 of *dupA *or at position 2998 of the *jhp0917*-*jhp0918 *gene of the J99 strain that created a premature stop codon and may have considerable effects on protein expression or function. In their study, they counted the truncated samples as *dupA*-positive; however, it is clear that these mutated sequences would not produce intact DupA protein. For example, the expression of the blood group antigen binding adhesin (BabA) protein does not always correlated with the *babA *gene expression [27]. It will be better to detect intact *dupA *by measuring intact DupA protein using immunoblotting techniques, which has not been reported previously. In addition, the *vir *genes exist before and after the region of the *dupA *locus [16]. In the strain Shi470, for example, *virB2, virB3, virB4 (dupA), virB8, virB9, virB10, virB11, virD4*, and *virD2 *were detected. These are structurally similar to the type IV secretion system (T4SS) called *cag *PAI and ComB and thought to be the third T4SS. Recently, T4SS containing *dupA *was named as *tfs3a*, and T4SS having *virB4 *sequence, but not *dupA *was named as *tfs3b *[28]. These observations suggest that only strains that are intact *dupA*-positive and form a novel type IV secretion system might be involved in gastroduodenal diseases. If this is true, examining the presence of DupA/*dupA *alone might not be sufficient. Study on DupA is still in their early stages, and great progress is expected in the near future.

## Conclusions

Infection with the *dupA*-positive *H. pylori *increased the risk for DU overall and this evidence was significant in Asian countries. In contrast, the relationship between *dupA *and GC and GU was not clear from the meta-analysis. Case-control study matched age- and sex- should be performed in the future.

## Competing interests

The authors declare that they have no competing interests.

## Authors' contributions

SS and YY designed the study, reviews potentially relevant articles, and performed the statistical analysis and wrote the manuscript. OM and MW helped to collect and interpret relevant articles and making figures. KH participated in the design of the study and helped to draft the manuscript. All authors read the final version of the manuscript.

## Financial support

None

## References

[B1] Suerbaum S Michetti P *Helicobacter pylori *infection N Engl J Med 2002 347 1175 1186 10.1056/NEJMra020542 12374879

[B2] Schistosomes, liver flukes and *Helicobacter pylori*. IARC Working Group on the Evaluation of Carcinogenic Risks to Humans. Lyon, 7-14 June 1994 IARC Monogr Eval Carcinog Risks Hum 1994 61 1 241 7715068 PMC7681621

[B3] Atherton J Cao P Peek RJ Tummuru M Blaser M Cover T Mosaicism in vacuolating cytotoxin alleles of *Helicobacter pylori*. Association of specific *vacA *types with cytotoxin production and peptic ulceration J Biol Chem 1995 270 17771 17777 10.1074/jbc.270.30.17771 7629077

[B4] Basso D Zambon C Letley D Stranges A Marchet A Rhead J Schiavon S Guariso G Ceroti M Nitti D Clinical relevance of *Helicobacter pylori cagA *and *vacA *gene polymorphisms Gastroenterology 2008 135 91 99 10.1053/j.gastro.2008.03.041 18474244

[B5] Gerhard M Lehn N Neumayer N Borén T Rad R Schepp W Miehlke S Classen M Prinz C Clinical relevance of the *Helicobacter pylori *gene for blood-group antigen-binding adhesin Proc Natl Acad Sci USA 1999 96 12778 12783 10.1073/pnas.96.22.12778 10535999 PMC23096

[B6] Yamaoka Y Kikuchi S el-Zimaity H Gutierrez O Osato M Graham D Importance of *Helicobacter pylori oipA *in clinical presentation, gastric inflammation, and mucosal interleukin 8 production Gastroenterology 2002 123 414 424 10.1053/gast.2002.34781 12145793

[B7] Lu H Hsu P Graham D Yamaoka Y Duodenal ulcer promoting gene of *Helicobacter pylori* Gastroenterology 2005 128 833 848 10.1053/j.gastro.2005.01.009 15825067 PMC3130061

[B8] Argent R Burette A Miendje Deyi V Atherton J The presence of *dupA *in *Helicobacter pylori *is not significantly associated with duodenal ulceration in Belgium, South Africa, China, or North America Clin Infect Dis 2007 45 1204 1206 10.1086/522177 17918084

[B9] Douraghi M Mohammadi M Oghalaie A Abdirad A Mohagheghi M Hosseini M Zeraati H Ghasemi A Esmaieli M Mohajerani N *dupA *as a risk determinant in *Helicobacter pylori *infection J Med Microbiol 2008 57 554 562 10.1099/jmm.0.47776-0 18436587

[B10] Gomes L Rocha G Rocha A Soares T Oliveira C Bittencourt P Queiroz D Lack of association between *Helicobacter pylori *infection with *dupA*-positive strains and gastroduodenal diseases in Brazilian patients Int J Med Microbiol 2008 298 223 230 10.1016/j.ijmm.2007.05.006 17897881

[B11] Schmidt H Andres S Kaakoush N Engstrand L Eriksson L Goh K Fock K Hilmi I Dhamodaran S Forman D Mitchell H The prevalence of the duodenal ulcer promoting gene (*dupA*) in *Helicobacter pylori *isolates varies by ethnic group and is not universally associated with disease development: a case-control study Gut Pathog 2009 1 5 10.1186/1757-4749-1-5 19338650 PMC2667403

[B12] Nguyen L Uchida T Tsukamoto Y Kuroda A Okimoto T Kodama M Murakami K Fujioka T Moriyama M *Helicobacter pylori dupA *gene is not associated with clinical outcomes in the Japanese population Clin Microbiol Infect 2010 16 1264 1269 10.1111/j.1469-0691.2009.03081.x 19832706

[B13] Hussein N The association of *dupA *and *Helicobacter pylori*-related gastroduodenal diseases Eur J Clin Microbiol Infect Dis 2010 29 817 821 10.1007/s10096-010-0933-z 20419465

[B14] Egger M Davey Smith G Schneider M Minder C Bias in meta-analysis detected by a simple, graphical test BMJ 1997 315 629 634 9310563 10.1136/bmj.315.7109.629 PMC2127453

[B15] Matysiak-Budnik T Laszewicz W Lamarque D Chaussade S *Helicobacter pylori *and non-malignant diseases Helicobacter 2006 11 Suppl 1 27 31 10.1111/j.1478-405X.2006.00426.x 16925608

[B16] Yamaoka Y Roles of the plasticity regions of *Helicobacter pylori *in gastroduodenal pathogenesis J Med Microbiol 2008 57 545 553 10.1099/jmm.0.2008/000570-0 18436586 PMC2833349

[B17] Matteo M Armitano R Granados G Wonaga A Sánches C Olmos M Catalano M *Helicobacter pylori oipA*, *vacA *and *dupA *genetic diversity in individual hosts J Med Microbiol 2010 59 89 95 10.1099/jmm.0.011684-0 19643933

[B18] Hussein N Argent R Marx C Patel S Robinson K Atherton J *Helicobacter pylori dupA *is polymorphic, and its active form induces proinflammatory cytokine secretion by mononuclear cells J Infect Dis 2010 202 261 269 10.1086/653587 20533870

[B19] Schmidt H Andres S Nilsson C Kovach Z Kaakoush N Engstrand L Goh K Fock K Forman D Mitchell H The *cag *PAI is intact and functional but *HP0521 *varies significantly in *Helicobacter pylori *isolates from Malaysia and Singapore Eur J Clin Microbiol Infect Dis 2010 29 439 451 10.1007/s10096-010-0881-7 20157752

[B20] Arachchi H Kalra V Lal B Bhatia V Baba C Chakravarthy S Rohatgi S Sarma P Mishra V Das B Ahuja V Prevalence of duodenal ulcer-promoting gene (*dupA*) of *Helicobacter pylori *in patients with duodenal ulcer in North Indian population Helicobacter 2007 12 591 597 10.1111/j.1523-5378.2007.00557.x 18001398

[B21] Hussein N Mohammadi M Talebkhan Y Doraghi M Letley D Muhammad M Argent R Atherton J Differences in virulence markers between *Helicobacter pylori *strains from Iraq and those from Iran: potential importance of regional differences in *H. pylori*-associated disease J Clin Microbiol 2008 46 1774 1779 10.1128/JCM.01737-07 18353934 PMC2395113

[B22] Yeh Y Cheng H Chang W Yang H Sheu B Matrix metalloproteinase-3 promoter polymorphisms but not *dupA*-*H. pylori *correlate to duodenal ulcers in *H. pylori*-infected females BMC Microbiol 2010 10 218 10.1186/1471-2180-10-218 20707923 PMC2928200

[B23] Pacheco A Proença-Módena J Sales A Fukuhara Y da Silveira W Pimenta-Módena J de Oliveira R Brocchi M Involvement of the *Helicobacter pylori *plasticity region and *cag *pathogenicity island genes in the development of gastroduodenal diseases Eur J Clin Microbiol Infect Dis 2008 27 1053 1059 10.1007/s10096-008-0549-8 18560912

[B24] Zhang Z Zheng Q Chen X Xiao S Liu W Lu H The *Helicobacter pylori *duodenal ulcer promoting gene, *dupA *in China BMC Gastroenterol 2008 8 49 10.1186/1471-230X-8-49 18950522 PMC2584642

[B25] Falush D Wirth T Linz B Pritchard J Stephens M Kidd M Blaser M Graham D Vacher S Perez-Perez G Traces of human migrations in *Helicobacter pylori *populations Science 2003 299 1582 1585 10.1126/science.1080857 12624269

[B26] Linz B Balloux F Moodley Y Manica A Liu H Roumagnac P Falush D Stamer C Prugnolle F van der Merwe S An African origin for the intimate association between humans and *Helicobacter pylori* Nature 2007 445 915 918 10.1038/nature05562 17287725 PMC1847463

[B27] Yamaoka Y Roles of *Helicobacter pylori *BabA in gastroduodenal pathogenesis World J Gastroenterol 2008 14 4265 4272 10.3748/wjg.14.4265 18666312 PMC2731175

[B28] Kersulyte D Lee W Subramaniam D Anant S Herrera P Cabrera L Balqui J Barabas O Kalia A Gilman R Berg D *Helicobacter Pylori*'s plasticity zones are novel transposable elements PLoS One 2009 4 e6859 10.1371/journal.pone.0006859 19727398 PMC2731543

